# The Presence of Pretreated Lignocellulosic Solids from Birch during *Saccharomyces cerevisiae* Fermentations Leads to Increased Tolerance to Inhibitors – A Proteomic Study of the Effects

**DOI:** 10.1371/journal.pone.0148635

**Published:** 2016-02-05

**Authors:** Rakesh Koppram, Valeria Mapelli, Eva Albers, Lisbeth Olsson

**Affiliations:** Industrial Biotechnology, Department of Chemical and Biological Engineering, Chalmers University of Technology, Gothenburg, Sweden; National Renewable Energy Lab, UNITED STATES

## Abstract

The fermentation performance of *Saccharomyces cerevisiae* in the cellulose to ethanol conversion process is largely influenced by the components of pretreated biomass. The insoluble solids in pretreated biomass predominantly constitute cellulose, lignin, and -to a lesser extent- hemicellulose. It is important to understand the effects of water-insoluble solids (WIS) on yeast cell physiology and metabolism for the overall process optimization. In the presence of synthetic lignocellulosic inhibitors, we observed a reduced lag phase and enhanced volumetric ethanol productivity by *S*. *cerevisiae* CEN.PK 113-7D when the minimal medium was supplemented with WIS of pretreated birch or spruce and glucose as the carbon source. To investigate the underlying molecular reasons for the effects of WIS, we studied the response of WIS at the proteome level in yeast cells in the presence of acetic acid as an inhibitor. Comparisons were made with cells grown in the presence of acetic acid but without WIS in the medium. Altogether, 729 proteins were detected and quantified, of which 246 proteins were significantly up-regulated and 274 proteins were significantly down-regulated with a fold change≥1.2 in the presence of WIS compared to absence of WIS. The cells in the presence of WIS up-regulated several proteins related to cell wall, glycolysis, electron transport chain, oxidative stress response, oxygen and radical detoxification and unfolded protein response; and down-regulated most proteins related to biosynthetic pathways including amino acid, purine, isoprenoid biosynthesis, aminoacyl-tRNA synthetases and pentose phosphate pathway. Overall, the identified differentially regulated proteins may indicate that the likelihood of increased ATP generation in the presence of WIS was used to defend against acetic acid stress at the expense of reduced biomass formation. Although, comparative proteomics of cells with and without WIS in the acetic acid containing medium revealed numerous changes, a direct effect of WIS on cellular physiology remains to be investigated.

## Introduction

Lignocellulosic biomass, a renewable non-food resource, is receiving increasing attention as a possible source of mixed carbohydrates for the biotechnological production of biofuels such as bioethanol. The structural organization of lignocellulosic biomass is robust and complex, hence, a pretreatment step is required to open the structure so that it becomes accessible to cellulolytic and hemicellulolytic enzymes, thus allowing efficient conversion of cellulose and hemicellulose into their respective monomers [1]. The pretreatment of lignocellulosic biomass produces slurry containing soluble and insoluble components. The soluble components include sugar monomers and oligomers derived from cellulose and hemicelluose, phenolic compounds derived from lignin, furan derivatives that are the degradation products of sugar monomers and weak acids such as acetic acid (which is an integral part of hemicellulose), formic and levulinic acid that are further derived from furans [2]. Insoluble components mainly include cellulose, lignin, and hemicellulose. The pretreated slurry can either be enzymatically hydrolyzed first and then subjected to microbial transformation in a separate hydrolysis and fermentation (SHF) process, or enzymatic hydrolysis of cellulose/hemicellulose in the slurry to sugar monomers can simultaneously occur with the fermentation of sugars to ethanol in a simultaneous saccharification and fermentation (SSF) process [3]. In either of these process configurations, the components of the slurry significantly influence the cellular physiology and metabolism of the fermenting microorganism, thereby, affecting the rates and yields of the desired product.

For the production of low-value and high-volume commodity chemicals such as ethanol, even minute levels of improvement in the rates and yields would significantly improve the overall economy of the process. As many companies are investing in the infrastructure, process development and production facilities for cellulosic ethanol production, it is crucial to understand how the whole substrate and the components of the substrate affect the performance of the fermenting microorganism, *Saccharomyces cerevisiae*.

In recent years, there has been significant progress in our understanding of the effects of soluble components of the slurry, such as lignocellulosic inhibitors on *S*. *cerevisiae* [4,5]and there are a number of strategies to improve the robustness of the fermenting microorganism [6–8]. Other soluble components of the slurry that have received much attention in recent years are the pentose sugars xylose and arabinose, which the wild-type *S*. *cerevisiae* cannot metabolize. Efforts have been made to develop *S*. *cerevisiae* strains that consume pentose sugars [9–11], and to further improve the efficiency of fermentation of pentose sugars to ethanol [12–14].

Another significant part of the pretreated biomass is made of insoluble solids or water-insoluble solids (WIS) which can collectively represent 10–35% (w/w) of the slurry. A large effort has been made in recent years to characterize the WIS and to understand the mechanisms of interaction of cellulose, hemicellulose, and lignin with the enzymes that act on these polymers [15–17]. However, there is currently no information available on the effects that WIS have on the fermentation performance of *S*. *cerevisiae*. Understanding the effects of WIS alone or in the presence of soluble inhibitors on cellular physiology would pave the way for further improvement of the robustness of *S*. *cerevisiae*. The effect of WIS can likely be regarded as one of the factors for consideration when choosing the process configuration for ethanol production.

In the present study, we found that in the presence of inhibitors, the fermentation performance of *S*. *cerevisiae* CEN.PK113-7D-in particular in terms of decrease of lag phase time and increase of volumetric ethanol productivity-was observed when WIS were present rather than absent. There appears to be a positive effect of insoluble solids on cellular performance, and we call this the ‘water-insoluble solids effect’ (WISE). This effect was observed when WIS of pretreated hardwood such as birch wood or pretreated softwood such as spruce wood were added to medium containing one or more of the following inhibitors: hydroxymethyl furfural (HMF), furfural, acetic acid, and syringaldehyde. In order to understand the physiological basis of WISE, we investigated differences in the protein expression profile of yeast grown under acetic acid stress in the presence and in the absence of washed WIS derived from pretreated birch wood.

## Materials and Methods

### Microorganism

The *S*. *cerevisiae* CEN.PK113-7D strain (kindly provided by Dr. Peter Kötter, Biozentrum, Frankfurt, Germany) was used in all experiments. The strain was stored at -80°C in culture aliquots containing 20% sterile glycerol. Volumes of 100 μl from the vials were used to inoculate precultures.

### Medium and fermentation conditions

Fermentation experiments in 50 ml centrifuge tubes were carried out in minimal medium containing 2% (w/w) glucose and enriched with salts, and two folds of trace elements and vitamins according to Verduyn *et al*. [18]. This medium was also used for inoculum development, which was carried out in 100 ml shake flasks to a working volume of 50 ml. According to the experimental requirement, whenever necessary, HMF and furfural, both to a final concentration of 0.1% (w/w); syringaldehyde to a final concentration of 0.08% (w/w); and acetic acid in concentrations of 0.2, 0.45, 0.75, 0.9% (w/w) were added to the minimal medium. The pH of the medium was adjusted to 5.7 with 5 M NaOH. The medium was inoculated to an initial OD_650_ of 0.01 and incubated at 30°C. The centrifuge tubes used for the fermentation experiments with WIS and its corresponding control experiments without WIS were fastened to a rotator (Stuart rotator SB3, Staffordshire, UK) set at 30 rpm that provided a free fall mixing [19] regime suitable at high solids concentrations. Whereas, mixing during the incubation of precultures at 30°C was performed in shake flasks in an orbital shaker set at 180 rpm. The fermentation experiments, carried out for the extraction of total intracellular proteome, were performed in triplicates. All other fermentation experiments were performed in duplicates. The data presented were mean of the replicates with error bars indicating the standard deviation.

### Birch WIS

Birch slurry was obtained from SEKAB E-Technology AB (Örnsköldsvik, Sweden) and it was stored at -20°C. The birch wood had been pretreated at 185°C for 5 min with 0.6% dilute sulfurous acid (sulphur dioxide in water). The composition of pretreated birch wood can be found in [20]. The solid fraction and liquid fraction of the resulting slurry were separated by centrifugation for 10 min at 10,000 *g*. The WIS was obtained by repeated washing of the solid fraction and centrifugation for 10 min at 10,000 *g* to remove monomeric and oligomeric sugars and soluble inhibitory compounds. Different concentrations of WIS (2, 5, 10, and 12% (w/w)) were added to the minimal medium where required.

### Protein extraction for proteomic analysis

The cells grown in minimal medium containing 7.5 g l^-1^ of acetic acid, both in the presence and absence of WIS, were harvested in the late exponential phase by centrifugation at 3,500 *g*, 4°C for 2 min. The cell pellets, with and without WIS, were washed with ice cold milliQ water. Acid washed glass beads, in amounts equal to the weight of the pellets, were added to the tubes containing the cell pellets. The tubes were filled with triethylammoniumbicarbonate (TEAB) buffer to a final concentration of 250 mM. The cells were lysed using a cell disrupter (FastPrep-24, MP Biomedicals, France) set at speed 6.0 for 20 seconds. The disrupter was run for six times with samples left on ice for 30 seconds between each run. After the cell disruption, sodium dodecyl sulfate (SDS) and dithiothreitol (DTT) were added to a final concentration of 4% (w/v) and 100 mM, respectively. The samples were heated at 60°C for 5 min and the supernatant was collected by centrifugation at the maximum speed for 2 min. The supernatant was centrifuged again to ensure residual particles were removed. The sample solution was purified through a particle filter (0.2 μm pore size) and then concentrated using 3K molecular weight cut-off filter in a centrifuge. Proteins were precipitated with acetone and the pellets dissolved in SDT buffer (250 mM triethyl ammonium bicarbonate (TEAB), 4% (w/v) SDS, and 100 mM dithiothreitol (DTT)). Total protein concentration was determined with the Pierce™ 660 nm Protein Assay (Thermo Scientific, Basel, Switzerland).

### Tryptic digestion and TMT (tandem mass tags) labelling of proteins

Protein extracts from each sample were trypsin-digested using the filter-aided sample preparation (FASP) method [21]. In short, samples were applied to Nanosep 10k Omega filters, (Pall Life Sciences, NY, USA) and 8M urea in 250 mM triethylammonium bicarbonate (TEAB) was used to repeatedly wash away the SDS. Trypsin (sequencing-grade modified trypsin; Promega, WI, USA) at a ratio of 1:25 relative to the amount of protein was added in 500 mM TEAB at a pH of about 8 and the samples were incubated for 16 h at 37°C. An additional portion of trypsin, same amount as the first addition, was freshly added and the mixture was further incubated for 3 h at 37°C. After centrifugation, the filtrates were subjected to isobaric mass tagging reagent TMT^®^ according to the manufacturer’s instructions (Thermo Scientific, MA, USA). Each biological replicate in a TMT 6-plex set was labelled with a unique tag. After TMT labelling, the samples were pooled and prepared for LC-MS/MS with SCX purification spin columns, HiPPR detergent removal, and PepClean C18 spin columns (all from Pierce Biotechnology), according to the instructions of the manufacturer.

The samples were dried down to approximately 50μl in a Speedvac. For the SCX fractionation the salt steps were increased automatically using a HPLC system and the UV absorbance was monitored to track the peptide containing fractions. Each fraction is automatically collected The 20 peptide-containing fractions were desalted using PepClean C18 spin columns (Thermo Fisher Scientific, Waltham, MA, USA) according to the manufacturer’s instructions.

### LC-MS/MS analysis on LTQ-Orbitrap Velos

The dried TMT-labelled peptides were reconstituted with 15 μl of 0.1% (v/w) formic acid (Sigma Aldrich, St Louis, MO, USA) in 3% (v/w) acetonitrile and analyzed on an LTQ-Orbitrap Velos mass spectrometer interfaced with an Easy-nLC II (Thermo Fisher Scientific, Waltham, MA, USA). Peptides (2 μl injection volume) were separated using an in-house constructed pre-column and analytical column set up (45 x 0.075 mm I.D. and 200 x 0.075 mm I.D., respectively) packed with 3 μm Reprosil-Pur C18-AQ particles (Dr. Maisch GmbH, Ammerbuch, Germany). The following gradient was run at 200 nl/min: 7–27% B-solvent (acetonitrile in 0.2% formic acid) over 60 min, 27–40% B-solvent over 10 min, 40–80% B-solvent over 10 min with a final hold at 80% B-solvent for 10 min. A second LC-MS/MS analysis with an extended gradient was also run: 5–10% B-solvent (acetonitrile in 0.2% formic acid) over 20 min, 10–25% B-solvent over 120 min, 25–40% B-solvent over 20 min, 40–80% B-solvent over 20 min with a final hold at 80% B-solvent for 10 min. Ions were injected into the LTQ-Orbitrap Velos mass spectrometer under a spray voltage of 1.6 kV in positive-ion mode. For MS scans, 1 microscan was performed at 30,000 resolution (full width at half maximum, FWHM, at *m/z* 400) over the *m/z* range 400–1,800. MS/MS analysis was performed in a data-dependent mode, with the top 10 most abundant doubly or multiply charged precursor ions in each MS scan selected for fragmentation (MS^2^) by stepped high-energy collision dissociation (HCD). For MS^2^ scans, 1 microscan was performed at 7,500 resolution (at *m/z* 400) with a mass range of *m/z* 100–2,000 using stepped normalized collision energies of 25%, 35%, and 45%. Dynamic exclusion for 30 s was used for *m/z* values already selected for fragmentation.

### Database search for protein TMT quantification

MS raw data files were merged for relative quantification and identification using Proteome Discoverer version 1.3 (Thermo Fisher Scientific, Waltham, MA, USA). A database search for each set was performed with the Mascot search engine (Matrix Science Ltd., London, UK) using a custom-made yeast protein database (protein sequence file of CEN.PK113-7D from *Saccharomyces* genome database) with MS peptide tolerance of 10 ppm and MS/MS tolerance of 100 millimass units (mmu). The Mascot significance threshold was set at 0.01, protein relevance threshold score at 20, resulting in expectation values for lowest ions score at 0.2. Tryptic peptides were accepted with one missed cleavage and variable modifications of methionine oxidation, cysteine methylthiolation, and fixed modifications of N-terminal TMT6plex and lysine TMT6plex were selected.

The detected peptide threshold in the software was set to 1% false discovery rate by searching against a reversed database [22,23], and proteins identified were grouped by sharing the same sequences to minimize redundancy. For TMT quantification, the ratios of the TMT reporter ion intensities in MS/MS spectra ([M+H]^+^ *m/z* 126–131) from raw data sets were used to calculate fold changes between samples. Ratios were derived with Proteome Discoverer using the following criteria: fragment ion tolerance = 100 mmu for the most confident centroid peak; TMT reagent purity corrections factors were used and missing values were replaced with minimum intensity. Only peptides unique for a given protein were considered for relative quantitation, excluding those common to other isoforms or proteins of the same family. The quantification was normalized using the protein median. The results were then exported to Excel for manual data interpretation, calculation of fold changes, and statistical analysis.

### Analysis of metabolites

Samples were analyzed for extracellular metabolites by high-performance liquid chromatography using a Rezex ROA-organic acid H+ (8%) column (Phenomenex Inc.) maintained at 80°C with 3 mm I.D. Carbo-H4 guard cartridge (Phenomenex Inc.) maintained at room temperature. As an eluent, 5 mM H_2_SO_4_ was used at a flow rate of 0.8 ml min^-1^. Glucose, glycerol, and ethanol were detected using an RI detector maintained at 35°C; and acetic acid, HMF, furfural, and syringaldehyde were detected using a UV detector at 210 nm.

### Data analysis, nLC-MS/MS

Fold change (FC) of the expression of each protein was calculated by dividing average ratio of each protein obtained from the yeast culture containing acetic acid in the presence of WIS with the average protein ratio in the yeast culture containing acetic acid without WIS. Fold change of less than 1 were inverted and given a negative sign. Student’s t-test was used for pairwise comparison. The resulting t-statistic values were compared with the value (degrees of freedom: 2) of 95% two-tailed t-test table and the proteins were sorted accordingly. The proteins were sorted again with the criterion of biological significant changes set to a |FC|≥1.2. See S1 Table for more information on t-test and protein sorting. The Saccharomyces Genome Database (http://www.yeastgenome.org/) was used as a reference to obtain information on proteins. Functional categorization and subcellular localization of proteins were carried out using FunCatDB software (http://mips.helmholtz-muenchen.de/funcatDB/).

## Results and Discussion

To the best of our knowledge, the effect of water-insoluble solids (WIS) resulting from pretreatment of lignocellulosic biomass on the fermentation performance of *S*. *cerevisiae* has not been reported previously. We evaluated the inhibitory effect of HMF, furfural, acetic acid, and syringaldehyde in the presence of WIS on CEN.PK113-7D strain. To understand the molecular physiology of cells exposed to WIS under acetic acid stress, a comparative proteomic investigation was performed.

### Water-insoluble solids improve the fermentation performance in the presence of inhibitors

**Fig 1:**
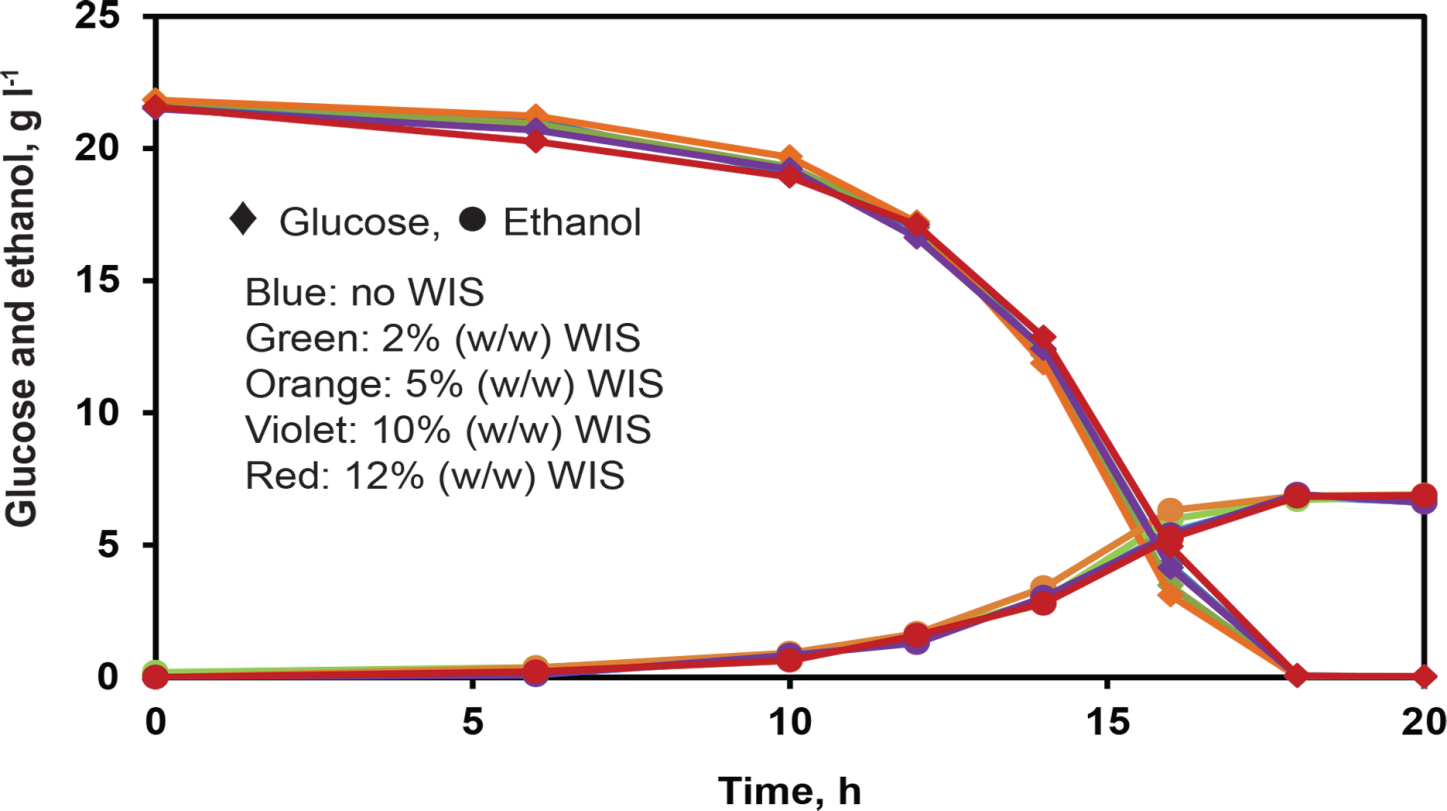
Influence of WIS on fermentation performance. Glucose consumption (diamonds) and ethanol production (circles) in minimal medium with 2% (w/w) glucose in the presence of no WIS (blue), 2% (w/w) WIS (green), 5% WIS (orange), 10% WIS (violet), and 12% WIS (red). Data represent the mean of biological duplicates. Error bars, representing the standard deviation, are smaller than the symbols to be visible.

The WIS of pretreated birch wood were washed with water to remove the soluble components in the slurry. The effect of WIS on the fermentation performance of strain CEN.PK113-7D was studied using minimal medium containing different concentrations of WIS (2, 5, 10 and 12% (w/w)). No differences in glucose consumption and ethanol production were observed in the control fermentation (with no WIS in the medium) compared to fermentations in the presence of WIS (Fig 1).

**Fig 2:**
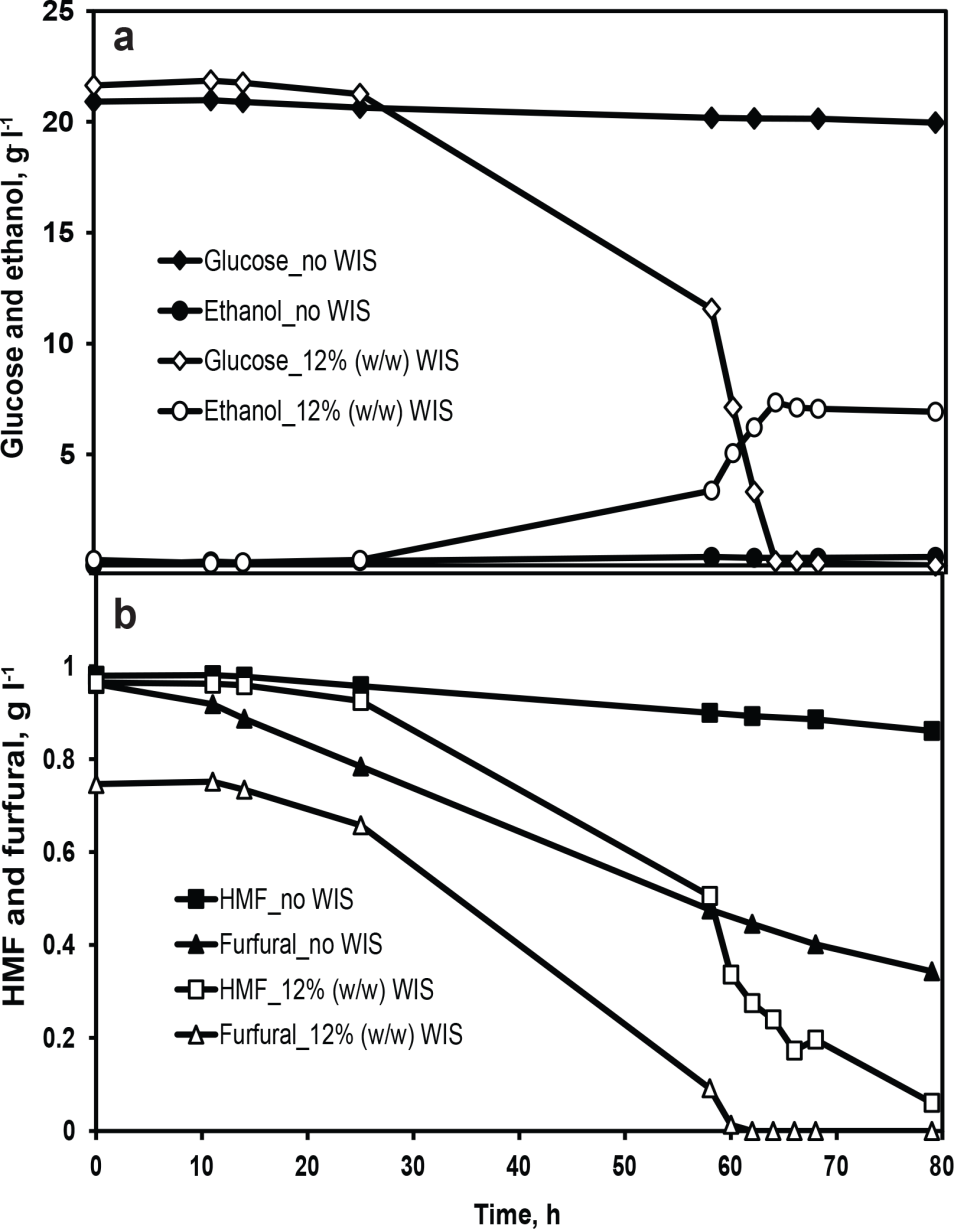
Influence of WIS on fermentation performance in the presence of 1 g l^-1^ HMF and 1 g l^-1^ furfural. (a) Glucose consumption (diamonds) and ethanol production (circles), (b) HMF (squares) and furfural (triangles) conversion in minimal medium with 2% (w/w) glucose in the presence of 12% (w/w) WIS (open symbols) and in control fermentation of no WIS (closed symbols). Data represent the mean of biological duplicates. Error bars, representing the standard deviation, are smaller than the symbols to be visible.

**Fig 3:**
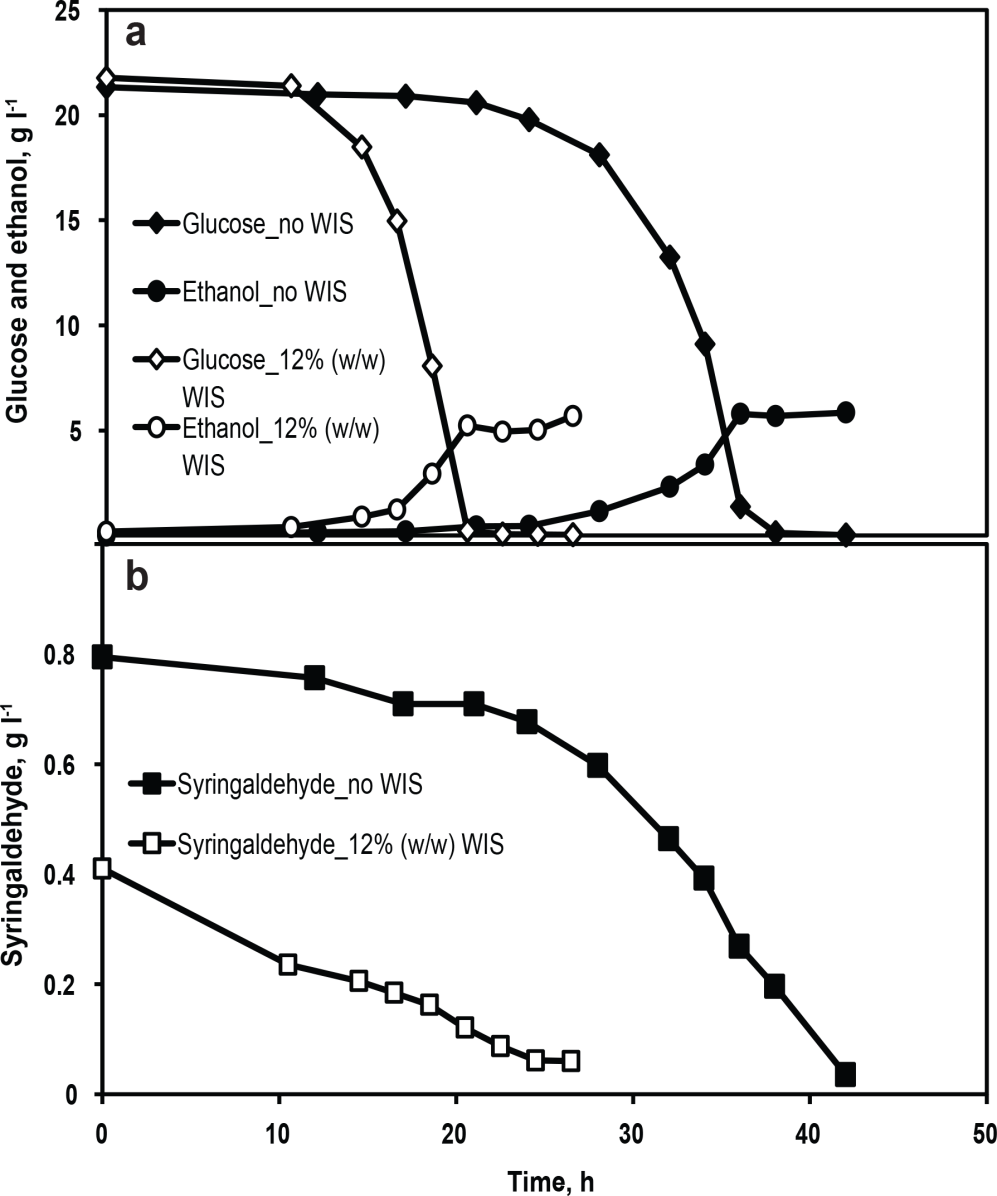
Influence of WIS on fermentation performance in the presence of 0.8 g l^-1^ syringaldehyde. (a) Glucose consumption (diamonds) and ethanol production (circles), (b) syringaldehyde conversion (squares) in minimal medium with 2% (w/w) glucose in the presence of 12% (w/w) WIS (open symbols) and in control fermentation of no WIS (closed symbols). Data represent the mean of biological duplicates. Error bars, representing the standard deviation, are smaller than the symbols to be visible.

**Fig 4:**
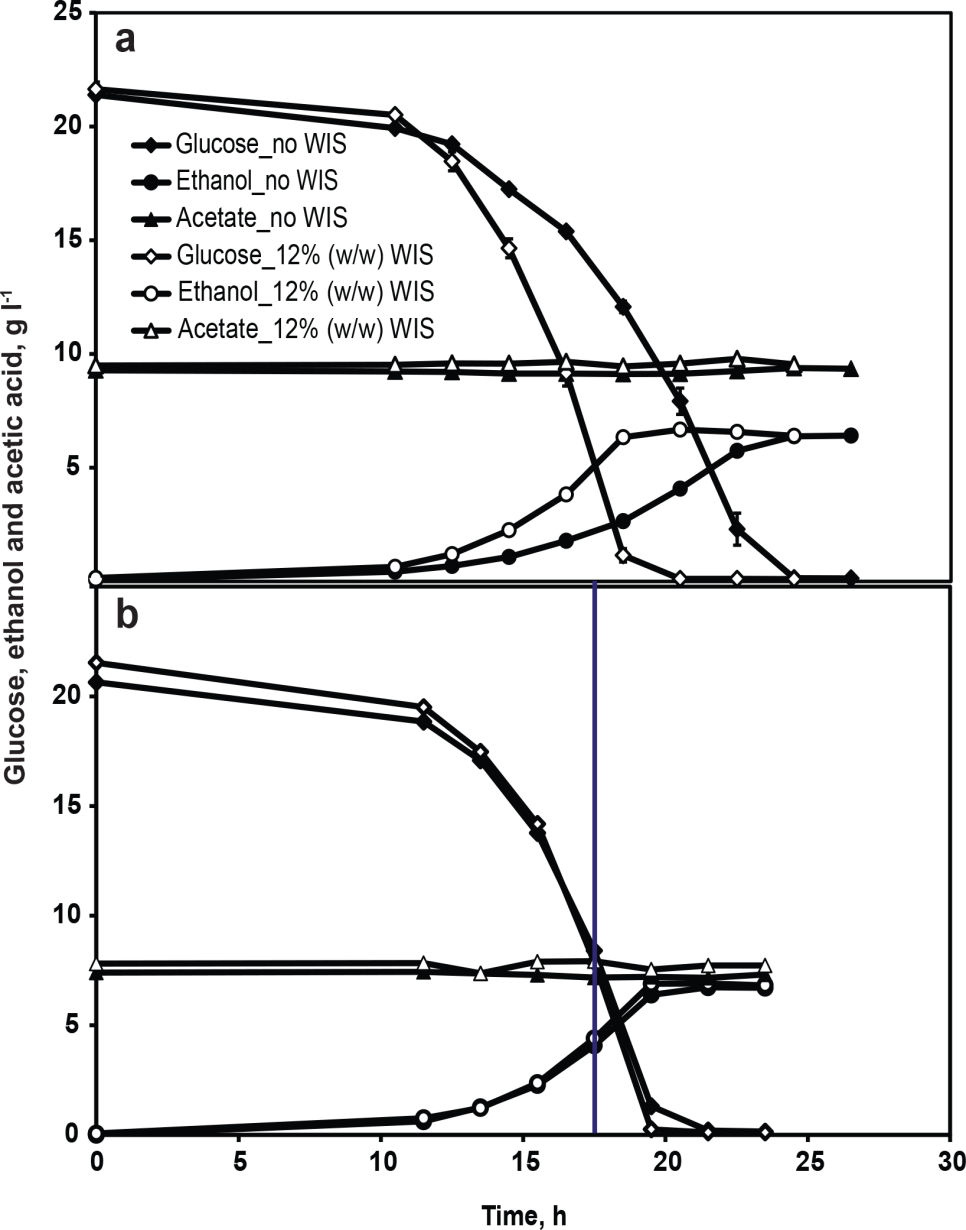
Influence of WIS on fermentation performance in the presence of acetic acid. Glucose consumption (diamonds), ethanol production (circles) and acetic acid conversion (triangles) in minimal medium with 2% (w/w) glucose in the presence of (a) 9 g l^-1^ of acetic acid and (b) acetic acid concentration of 7.5 g l^-1^ that was used for the proteome study and a straight line at 17.5 h indicates the time of sampling for the total protein extraction. Open and closed symbols represent experimental conditions in the presence of 12% (w/w) WIS and no WIS, respectively. Data in Fig 4a and 4b represent the mean of biological duplicates and triplicates, respectively. Error bars, representing the standard deviation, are smaller than the symbols to be visible.

Interestingly, there were significant differences in fermentation performance when lignocellulosic inhibitors were added to the minimal medium with WIS when compared to cultures without WIS addition. The concentration of WIS was 12% (w/w) in all the experiments unless, otherwise stated. HMF and furfural, syringaldehyde, and acetic acid were chosen as representative inhibitors from the inhibitor categories furans, phenolics, and weak acids, respectively. The effect of WIS on fermentation performance in the presence of inhibitors was evaluated. In the presence of 1 g l^-1^ HMF and 1 g l^-1^ furfural, no glucose consumption and ethanol production was observed for 80 h (Fig 2A) in the control fermentation (without WIS). In contrast, when WIS was present in minimal medium containing HMF and furfural, glucose was consumed completely within 65 h, yielding a maximum ethanol concentration of 7.4 g l^-1^ (Fig 2A). The conversion of HMF and furfural by the cells were relatively enhanced in the presence of WIS (Fig 2B). In the medium containing 0.8 g l^-1^ syringaldehyde, a lag phase of 24 h was observed in the control fermentation, but in the presence of WIS a lag phase of 14 h was observed and the glucose was completely consumed in 20.5 h (Fig 3A). It is noteworthy that the concentration of furfural or syringaldehyde measured in minimal medium containing WIS was lower than the added concentration (Fig 2B and Fig 3B), indicating a possible preferential adsorption of these inhibitors to the WIS. It can be hypothesized that a significant proportion of furfural or syringaldehyde was bound to the WIS, thereby reducing its bulk concentration and thus giving reduced levels of inhibition compared to the level of inhibition observed in the control fermentation. In order to partially verify this hypothesis, a highly water-soluble inhibitor such as acetic acid that has no adsorption affinity for WIS was chosen and its effect on fermentation performance was assessed. No significant difference was observed between the concentration of acetic acid measured and the concentration added to the medium containing WIS (Fig 4). In the presence of 9 g l^-1^ acetic acid (Fig 4A), the volumetric rates of glucose consumption and ethanol production were 1 and 0.34 g l^-1^ h^-1^, respectively, in the fermentation with WIS as compared to 0.87 and 0.26 g l^-1^ h^-1^ in the control fermentation (Table 1). The improved fermentation performance of CEN.PK113-7D in the presence of WIS strongly indicates that WIS has an influence on cellular physiology and metabolism. Thus, identification of the molecular factors responsible for WISE is crucial for further improvement of the robustness of a fermentation process. Also, in a wider perspective, the WISE could be considered to be one of the factors that decide the choice of process mode (i.e. Separate Hydrolysis and Fermentation vs. Simultaneous Saccharification and Fermentation) for cellulosic ethanol production.

**Table 1:** Yields and volumetric rates of fermentation in the presence of various concentrations of acetic acid.

Experimental condition	Y_ethanol/S,_ g g ^-1^	Y _glycerol/S_, g g^-1^	r_glucose_,g l^-1^ h^-1^	r_ethanol_, g l^-1^ h^-1^
MM_no WIS	0.32±0.00	0.06±0.00	1.20±0.00	0.38±0.00
MM_12% WIS	0.32±0.00	0.07±0.00	1.20±0.00	0.38±0.00
MM_Acetic acid (6.0 g l^-1^)	0.32±0.00	0.06±0.00	1.07±0.00	0.35±0.00
MM_Acetic acid (6.0 g l^-1^)_12% WIS	0.32±0.01	0.06±0.01	1.08±0.01	0.36±0.01
MM_Acetic acid (7.5 g l^-1^)	0.33±0.00	0.06±0.00	0.99±0.02	0.33±0.01
MM_Acetic acid (7.5 g l^-1^)_12% WIS	0.32±0.00	0.05±0.00	0.99±0.01	0.34±0.00
MM_Acetic acid (9.0 g l^-1^)	0.30±0.00	0.08±0.00	0.87±0.01	0.26±0.01
MM_Acetic acid (9.0 g l^-1^)_12% WIS	0.30±0.00	0.06±0.00	1.00±0.02	0.34±0.01

MM (minimal medium); data represents the mean and the values after ± indicates deviation from the mean.

### Proteins differentially expressed in the presence of WIS

Our choice of fermentation conditions for the proteome study was inclusion of acetic acid in the medium containing WIS, where there was an obvious influence of WIS on the fermentation performance. Acetic acid stress is known to reduce the specific growth rate [24,25] and changes in protein expression could arise from differences in specific growth rates. Thus, it was important for proteome analysis to determine a threshold concentration of acetic acid at which the specific growth rates would be same between the control fermentation and fermentation with WIS. However, determination of specific growth rate in the presence of WIS is a challenging task, since the cells are inseparable from the solids. As a consequence of this, determination of cell concentration either by optical density measurement or cell dry weight measurement becomes unreliable. We therefore assumed that the specific growth rate was identical between the two conditions if the volumetric rates of glucose consumption and ethanol production and final ethanol yields were virtually identical between the two conditions. Different concentrations of acetic acid were screened, and the highest concentration of acetic acid at which the volumetric rates of glucose consumption and ethanol production were virtually identical between the two conditions was found to be 7.5 g l^-1^ (Table 1 and Fig 4B), which was therefore chosen as a condition for fermentation and subsequent harvesting of cells for protein extraction. Protein extraction from biological triplicates of control fermentation and fermentation with WIS in the presence of 7.5 g l^-1^ acetic acid was done from the samples taken in late exponential growth phase at 17.5 h after inoculation (point of sampling is indicated by a straight line in Fig 4B). The protein extracts were further processed (as described in the Materials and methods) and subjected to nanoLC-MS/MS analysis.

**Fig 5:**
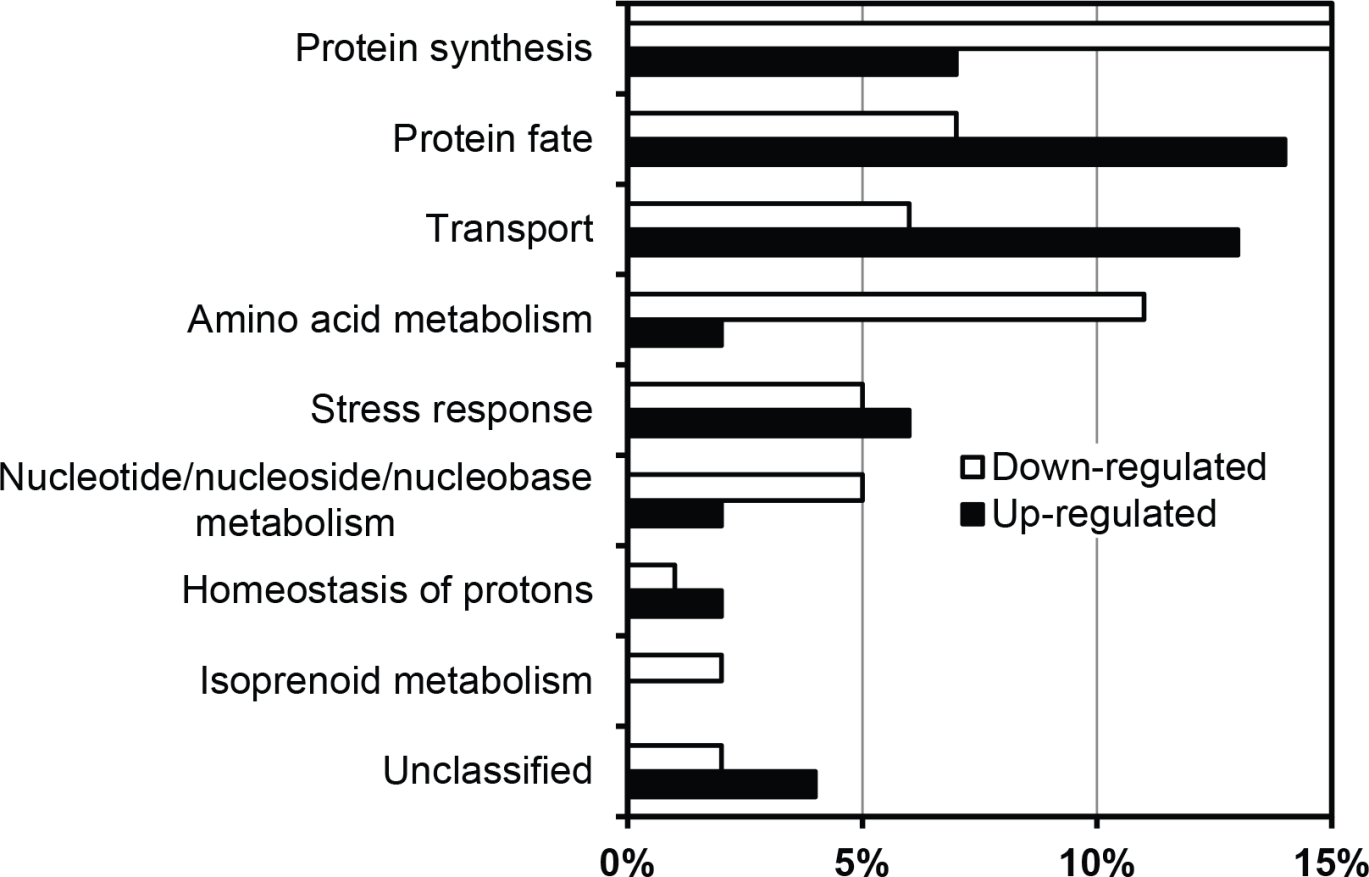
Functional categories of differentially expressed genes in the presence of WIS in medium containing acetic acid, based on MIPS indices of biological functions. The numbers of proteins in each category relative to the total number of differentially expressed proteins with a fold change of ≥ 1.2, expressed as percentage, are presented.

In total, 729 proteins were quantified (S1 Table) from the fermentation with WIS and from the control fermentation, both in the presence of acetic acid. On testing the protein quantification data with Student’s t-test with 95% confidence interval, 246 proteins were found to be up-regulated by 1.2 fold or more and 274 proteins were down-regulated by 1.2 fold or more when comparing the fermentation with WIS to the control fermentation. The differentially regulated proteins were functionally categorized using FunCatDB software (Fig 5, S2 Table). The major categories representing the up-regulated proteins were ‘protein fate’ categories covering proteins involved in folding, sorting, targeting, and protein/peptide degradation; and ‘cellular transport’ categories covering proteins involved in protein transport, electron transport, transport ATPases, mitochondrial, non-vesicular ER, and vacuolar transport. The major categories representing the down-regulated proteins in the presence of WIS were ‘protein synthesis’ categories covering proteins involved in ribosome biogenesis, translation, and aminoacyl-tRNA synthetases; ‘amino acid metabolism’ categories covering proteins involved in biosynthesis and metabolism of amino acids and sulphur metabolism; ‘C-compound and carbohydrate metabolism’ categories covering proteins involved in sugar, glucoside, polyol, and carboxylate catabolism, aromate anabolism, and transfer of activated C-1 groups; and ‘nucleotide/nucleoside/nucleobase metabolism’ categories covering proteins involved in purine nucleotide/nucleoside/nucleobase anabolism. The categories ‘energy’ and ‘stress response’ were represented in both the up- and down-regulated proteins–more or less equally. In the ‘energy’ category, the up- and down-regulated proteins were about equally distributed in the tricarboxylic-acid and pentose phosphate pathways, whereas the proteins involved in glycolysis, electron transport, and membrane- associated energy conservation and energy generation (ATP synthases) were up-regulated. In the ‘stress response’ category, the up- and down-regulated proteins were equally associated with the unfolded protein response (UPR), whereas the proteins involved in the oxidative stress response, and oxygen and radical detoxification, were up-regulated. ‘Isoprenoid metabolism’ stood out as a category represented exclusively in the dataset of down-regulated proteins. To a large extent, the categorization of differentially regulated proteins was in accordance with previously published results on genome-wide [26,27] and proteome-wide [28] screening in *S*. *cerevisiae* in response to acetic acid stress compared to no acetic acid stress. This clearly indicates that the presence of WIS alleviates acetic acid stress and further validates the improved fermentation performance of CEN.PK113-7D strain in the medium containing acetic acid in the presence of WIS (Fig 4A).

#### Influence of WIS on the cell wall and plasma membrane

We have identified some of the cell wall related proteins that are differentially regulated including Scw4p (3.8), Cis3p (3.2), Tos1p (2.8), Crh1p (2.9), Scw10p (2.5), Pst1p (1.9), Gas1p (1.9), Gas5p (1.5), Psa1p (-2.9), and Exg1p (-1.5). It has been shown that a double knockout of *SCW4* and *SCW10* in *S*. *cerevisiae* resulted in slower growth and increased release of proteins from intact cells by DTT treatment [29]. Another study has shown that cells with deletion of genes coding for Gas1p, Gas5p, Scw4p, Crh1p, Scw10p have altered cell wall properties [30]; Some of the proteins associated with co- and post-translational translocation of proteins, including Kar2p (1.4), Sbh1p (1.3), Sss1p (1.2), Ssa3p (1.4), Ssa4p (1.5), and Caj1p (1.5), and proteins associated with COPII vesicles that are responsible for transporting proteins from ER to Golgi including Sar1p (1.4) and Yip3p (1.4) were found to be up-regulated. The elevated levels of cell wall related proteins, proteins involved in co- and post-translational translocation of proteins, and ER to Golgi transporting proteins, that transport the plasma membrane proteins to their target location, may indicate that in the presence of solids the integrity of cell wall and plasma membrane can be challenged, forcing the cells to reorganize the cell wall and plasma membrane and the associated proteins that might be subjected to higher turnover [31].

#### Differentially regulated proteins indicate increased ATP generation and reduced biomass formation in the presence of WIS

The perturbation of proton homeostasis upon dissociation of acetic acid in the cytoplasm, due to higher intracellular pH compared to the extracellular environment, stimulates the activity of the plasma membrane H^+^ATPase (Pma1p)–a major regulator of cytoplasmic pH–to pump protons out of the cell at the expense of ATP [32]. Our proteome analysis showed down-regulation of Pma1p (-1.9) in the presence of WIS, suggesting that the protein was relatively abundant in the cells grown without WIS. This is already a clear indication that in the presence and absence of WIS, the cells were affected and/or responded differently to acetic acid in the medium.

It is also known that acetic acid and other lignocellulosic inhibitors elicit UPR [33]. We observed that several molecular chaperones and proteins with ATPase activity–or those that stimulate ATPase activity such as Hsp60p (1.5), Ssc1p (1.4), Rpt5p (1.4), Kar2p (1.4), Ssa3p (1.4), Rpt3p (1.2), and Hch1p (1.2), which are involved in protein folding, unfolding, translocation, and degradation–were up-regulated in the presence of WIS (S1 and S2 Tables). It can be inferred that in the presence of WIS, cells experience less intracellular acidification in the presence of acetic acid than cells in the control fermentation. Thus, in the presence of WIS cells may direct the cellular ATP more towards the protein folding/degradation machinery rather than the intracellular pH homeostasis process. Also, it has been found previously that Vacuolar H^+^ ATPases sequester H^+^ ions in the lumen and regulate intracellular pH. Our proteome analysis showed down-regulation of subunits of vacuolar H^+^ ATPases including Vma1p (-1.6), Vma5p (-1.7), and Vma8p (-1.3), but, the subunits Vma2p, Vma4p, and Vma7p–the deletion of which has been found to increase the sensitivity of cells to acids–[27] were up-regulated by 1.2, 1.4, and 1.8 fold, respectively, along with Vma10p (1.6).

Despite the differences in expression levels of proteins involved in the homeostasis of protons and the protein folding/degradation machinery, there is a clear demand for ATP in these processes. It has already been shown that the genes and proteins associated with glycolysis were over-expressed under acetic acid stress [26,28] in response to the demand for ATP. We observed that in the presence of WIS, cells up-regulated the glycolytic enzymes (S1A Fig) including Hxk2p (1.3), Pgi1p (1.3), Tpi1p (1.5), Tdh1p (1.5), Tdh2p (1.4), Pgk1p (1.4), Gpm1p (1.5), Gpm2p (1.2), and Eno1p (1.5), possibly indicating the response to the demand for ATP. We also observed increased activity of the electron transport chain; the up-regulation of proteins involved in the electron transport chain including Cox5ap (3.1), Cox4p (1.6), Cox6p (1.3), Qcr7p (1.5), and the subunits of F1F0 ATP synthases including Atp14p (1.7), Atp16p (1.7), Atp2p (1.6), Atp7p (1.6), Atp3p (1.3), Atp1p (1.2), and Atp18p (1.2) clearly indicates that when exposed to acetic acid and in the presence of WIS, cells regulate their metabolism more towards increased and more efficient ATP generation than when WIS are not present. It is well known that weak acids induce oxidative stress by increasing the production of free radicals by the electron transport chain [34]. In the presence of WIS, yeast cells significantly up-regulated the proteins responsible for oxygen and radical detoxification including Trx2p (2.2), Sod2p (1.8), Ahp1p (1.7), Trx1p (1.5), Sod1p (1.5), Grx3p (1.5), Grx5p (1.3), Tsa1p (1.2), and Grx1p (1.2), strongly indicating an increased counter-measure to oxidative stress.

The redox cofactor, NADH, produced in surplus amounts in the biosynthetic reactions is oxidized in the glycerol biosynthesis pathway. It has been shown that deletion of *GPD2*, one of the genes encoding glycerol 3-phosphate dehydrogenases of *S*. *cerevisiae*, resulted in reduced glycerol and biomass yields [35]. In this study, the down-regulation of enzymes involved in glycerol biosynthesis (Gpd2p (-1.5) and Rhr2p (-3.8)) was in accordance with the reduced glycerol yields with increasing acetic acid concentration in fermentation in the presence of WIS compared to control fermentation under acetic acid stress (Table 1). Together with the reduced glycerol yield and the down-regulation of enzymes involved in biosynthetic pathways including amino acid biosynthesis (S2 Fig), purine biosynthesis (S1B Fig), aminoacyl-tRNA synthases (S3A Fig), isoprenoid biosynthesis (S3B Fig) and pentose phosphate pathway (S3C Fig) may indicate reduced biomass formation in the presence of WIS under acetic acid stress. It can be speculated that the likelihood of increased generation of ATP, as discussed earlier, was used to regulate the protein folding/degradation machinery and intracellular pH at the expense of reduced biomass formation in the presence of WIS. The proteomic analysis in the present study is in accordance with the results of a recent proteomic analysis which showed that inhibitors tolerant yeast strain showed increased expression of glycolytic enzymes relative to its parental strain in order to produce more energy and reduced expression of proteins involved in nitrogen metabolism (amino acid and nucleotide metabolism)–to conserve energy in order to defend against inhibitors [33]. The evidence generated in this study indicates that in the presence of WIS, yeast cells are more tolerant to inhibitors than cells grown in the absence of WIS.

## Conclusions

The WIS (composed of cellulose, hemicellulose, and lignin) represent a significant proportion of the medium when performing fermentation using lignocellulosic materials at high dry matter content. We investigated the influence of WIS on fermentation performance, and found that the mere presence of WIS in the fermentation medium induced the cells to be more tolerant to lignocellulosic inhibitors compared to cells in the absence of WIS. Notably, in the presence of WIS the lag phase was significantly reduced and the volumetric rates of glucose consumption and ethanol production were significantly enhanced in medium containing furans, syringaldehyde, and acetic acid, compared to control fermentation without WIS. In the presence of acetic acid, proteomic analysis of WISE revealed up-regulation of proteins involved in the protein folding/degradation machinery, the oxidative stress response, and oxygen and radical detoxification; up-regulation of glycolytic enzymes and proteins related to the electron transport chain and ATP synthases strongly indicated that there was increased generation of energy in the presence of WIS than in the control fermentation. The majority of proteins related to biosynthetic pathways including amino acid biosynthesis, aminoacyl-tRNA synthetases, purine biosynthesis, isoprenoid biosynthesis, and pentose phosphate pathway were down-regulated in the presence of WIS, possibly indicating reduced energy expenditure and the channeling of energy to cope with acetic acid stress and the stress caused by the WIS on the integrity of cell wall and cell membrane. The latter stress can be hypothesized based on the observed up-regulation of proteins related to cell wall and plasma membrane and proteins involved in mechanisms of endocytosis and protein translocation typically linked to protein turnover processes. The stress caused by the WIS may presumably activate stress responses that prompts the cells to effectively respond to other stresses such as the one elicited by lignocellulosic inhibitors. Although comparative proteomics of cells with or without WIS in the acetic acid-containing medium revealed numerous changes, a direct effect of WIS on cellular physiology remains elusive and therefore, opens up a plethora of opportunities for further investigation.

## Supporting Information

S1 Fig **Differential expression of proteins involved in (a) glycolysis and the TCA cycle and (b) purine biosynthesis**. In comparison to the cells in the control fermentation, the protein levels elevated in the presence of WIS are indicated in red, the protein levels reduced in the presence of WIS are indicated in green, and protein levels showing no difference are indicated in grey. Boxes with no color indicate either the protein was not detected or protein level changes are not significant.
(TIF)

S2 Fig Differential expression of proteins involved in amino acid biosynthesis. In comparison to the cells in the control fermentation, the protein levels elevated in the presence of WIS are indicated in red, the protein levels reduced in the presence of WIS are indicated in green, and protein levels showing no difference are indicated in grey. Boxes with no color indicate either the protein was not detected or protein level changes are not significant.
(TIF)

S3 Fig **Differential expression of proteins involved in (a) aminoacyl-tRNA synthetases; (b) isoprenoid biosynthesis; and (c) the pentose phosphate pathway**. In comparison to the cells in the control fermentation, the protein levels elevated in the presence of WIS are indicated in red, the protein levels reduced in the presence of WIS are indicated in green, and protein levels showing no difference are indicated in grey. Boxes with no color indicate either the protein was not detected or protein level changes are not significant.
(TIF)

S1 Table List of identified proteins using nLC-MS/MS. (XLSX)

S2 Table Functional and localization categories of differentially regulated proteins. Proteins with a significance of 95% using Student’s t-test were categorized based on MIPS indices of biological functions and location. MIPS-generated P-value is also presented corresponding to each category. The proteins are designated according to the names of their ORFs.
(XLSX)
