# Supplementary figures and images for: The Presence of Pretreated Lignocellulosic Solids from Birch during Saccharomyces cerevisiae Fermentations Leads to Increased Tolerance to Inhibitors – A Proteomic Study of the Effects
Source: PLoS One. 2016 Feb 5;11(2):e0148635. doi: 10.1371/journal.pone.0148635 (PMC4743953; doi:10.1371/journal.pone.0148635)

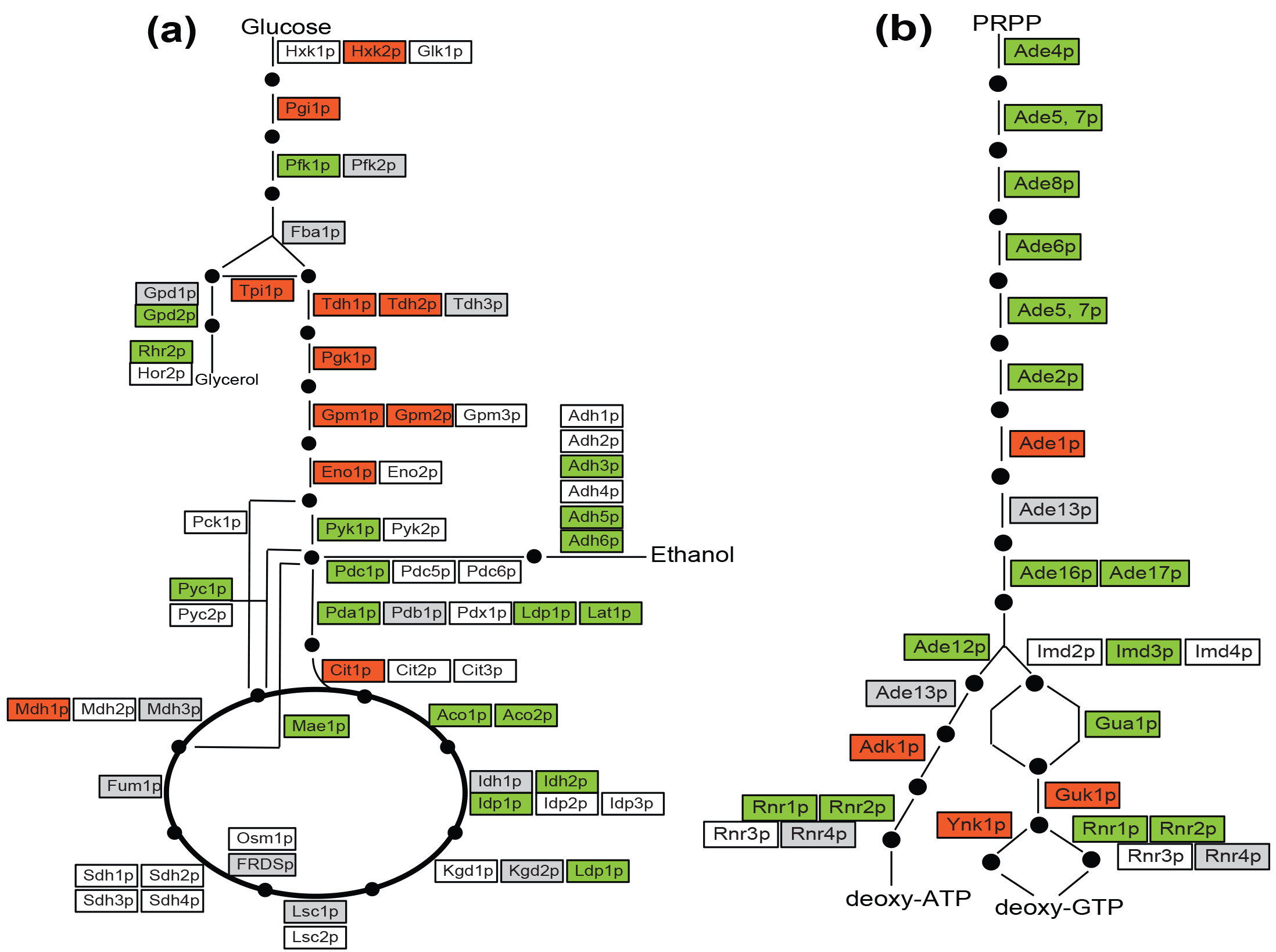

Supplement: S1 Fig — Differential expression of proteins involved in (a) glycolysis and the TCA cycle and (b) purine biosynthesis. In comparison to the cells in the control fermentation, the protein levels elevated in the presence of WIS are indicated in red, the protein levels reduced in the presence of WIS are indicated in green, and protein levels showing no difference are indicated in grey. Boxes with no color indicate either the protein was not detected or protein level changes are not significant. (TIF) [file pone.0148635.s001.tif]

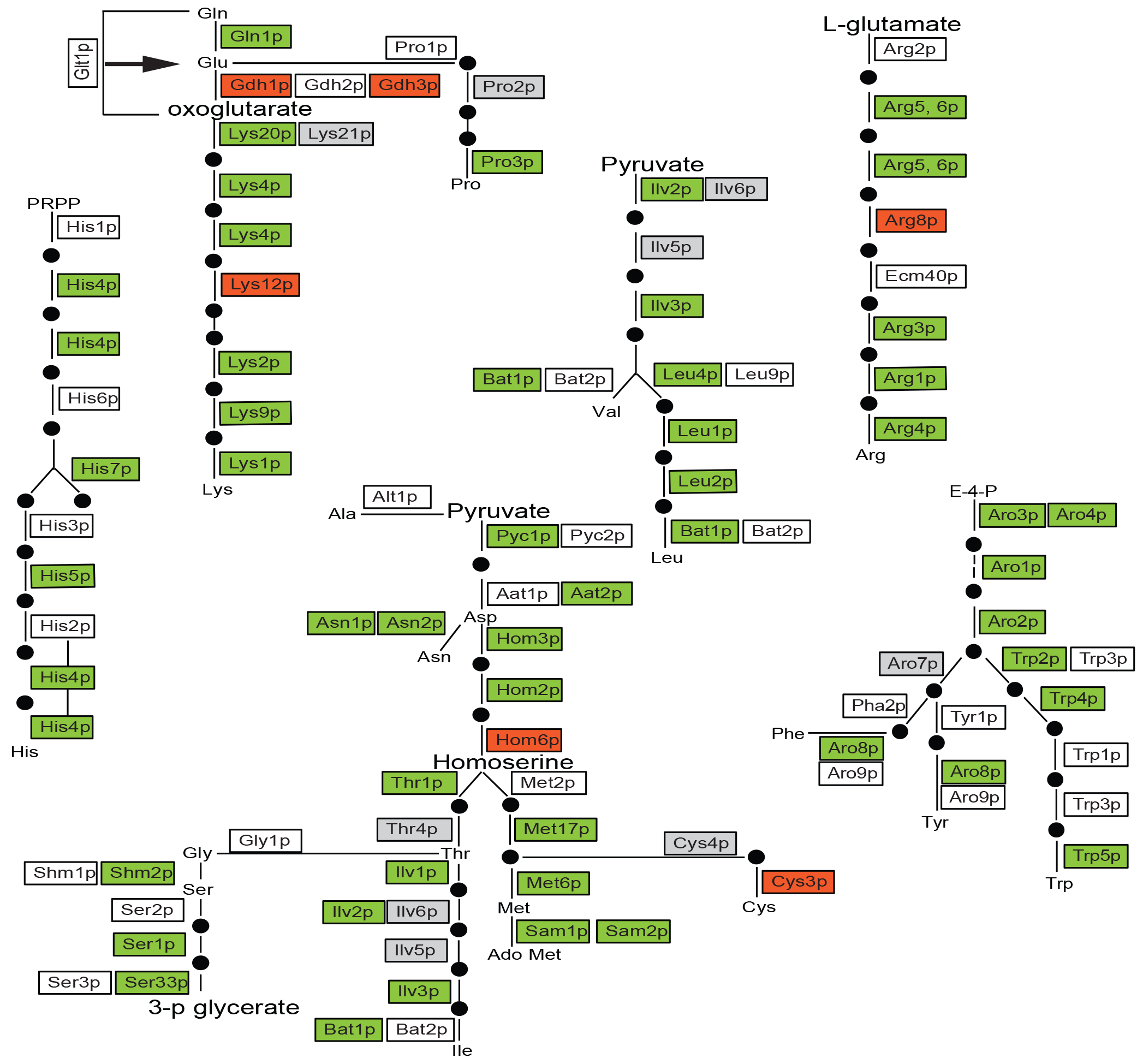

Supplement: S2 Fig — In comparison to the cells in the control fermentation, the protein levels elevated in the presence of WIS are indicated in red, the protein levels reduced in the presence of WIS are indicated in green, and protein levels showing no difference are indicated in grey. Boxes with no color indicate either the protein was not detected or protein level changes are not significant. (TIF) [file pone.0148635.s002.tif]

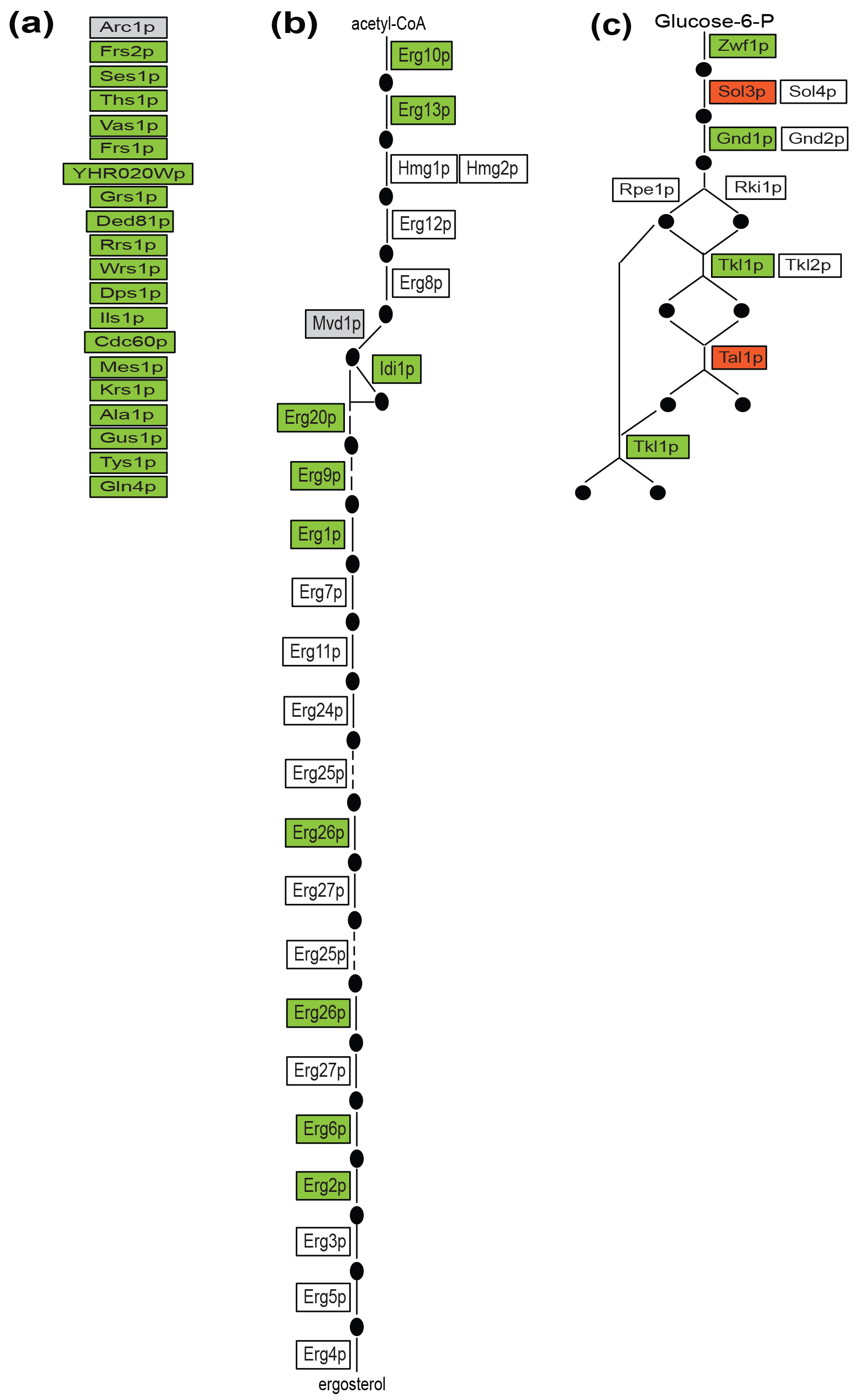

Supplement: S3 Fig — Differential expression of proteins involved in (a) aminoacyl-tRNA synthetases; (b) isoprenoid biosynthesis; and (c) the pentose phosphate pathway. In comparison to the cells in the control fermentation, the protein levels elevated in the presence of WIS are indicated in red, the protein levels reduced in the presence of WIS are indicated in green, and protein levels showing no difference are indicated in grey. Boxes with no color indicate either the protein was not detected or protein level changes are not significant. (TIF) [file pone.0148635.s003.tif]
